# RGB-D Object SLAM Using Quadrics for Indoor Environments

**DOI:** 10.3390/s20185150

**Published:** 2020-09-09

**Authors:** Ziwei Liao, Wei Wang, Xianyu Qi, Xiaoyu Zhang

**Affiliations:** Robotics Institute, Beihang University, Beijing 100191, China; liaoziwei@buaa.edu.cn (Z.L.); qixianyu@buaa.edu.cn (X.Q.); zhang_xy@buaa.edu.cn (X.Z.)

**Keywords:** semantic SLAM, object SLAM, quadrics, RGB-D, mobile robots, data association, nonparametric pose graph, indoor environments

## Abstract

Indoor service robots need to build an object-centric semantic map to understand and execute human instructions. Conventional visual simultaneous localization and mapping (SLAM) systems build a map using geometric features such as points, lines, and planes as landmarks. However, they lack a semantic understanding of the environment. This paper proposes an object-level semantic SLAM algorithm based on RGB-D data, which uses a quadric surface as an object model to compactly represent the object’s position, orientation, and shape. This paper proposes and derives two types of RGB-D camera-quadric observation models: a complete model and a partial model. The complete model combines object detection and point cloud data to estimate a complete ellipsoid in a single RGB-D frame. The partial model is activated when the depth data is severely missing because of illuminations or occlusions, which uses bounding boxes from object detection to constrain objects. Compared with the state-of-the-art quadric SLAM algorithms that use a monocular observation model, the RGB-D observation model reduces the requirements of the observation number and viewing angle changes, which helps improve the accuracy and robustness. This paper introduces a nonparametric pose graph to solve data associations in the back end, and innovatively applies it to the quadric surface model. We thoroughly evaluated the algorithm on two public datasets and an author-collected mobile robot dataset in a home-like environment. We obtained obvious improvements on the localization accuracy and mapping effects compared with two state-of-the-art object SLAM algorithms.

## 1. Introduction

Simultaneous localization and mapping (SLAM) has developed quickly in recent decades and has been widely used in fields such as autonomous driving, augmented reality, and robotics. The vision-based method has attracted widespread attention because cameras can capture rich details of the scene. The conventional visual SLAM extracts static geometric features in the environment, such as points, lines, and planes, and achieves high-precision localization and mapping [1]. Some also use the global appearance of whole images such as global-appearance descriptors [2,3], or gist descriptor [4] for mapping or localization, which model more information in an image compared with features.

However, considering a service robot that works in human-robot coexisting environments such as homes, museums, offices, some commands like “Move to the TV”, “Hand over the cup on the table” contain instructions to interact with objects in the environment, which requires the robot to have a semantic understanding about where the objects are. Instead of a specific coordinate point, we want the robot to understand and reach the target area. The robot needs to have an object concept, such as its semantic label, location, and roughly occupied space. It may also need to know orientation information to wait in the side of a chair and avoid standing in the human’s way. Therefore, a map that only contains geometric features in the environment is far from meeting the demand.

A service robot needs to work for a long time in the work scene, which arises the study of life-long robots [5]. A life-long robot needs to adapt to illumination changes, sensor noise, and significant viewing angle changes—all of those put forward requirements for the robustness of landmarks in its map. The existing visual SLAM technology relies on feature descriptors [6,7], surface textures [8,9], or the geometric position of plane and line structures [10,11,12] to solve data associations. However, artificially designed descriptors are challenging to adapt to significant viewing angle changes and are easily disturbed by illumination and sensor noise. Besides, the robot needs to adapt to long-term environmental changes, such as chairs, teacups, and other objects placed at will, new furniture added or removed. Maps based on point, line, and plane features are difficult to update directly, while an object-centric map can provide much convenience.

We propose that as essential components of the indoor environment, objects have geometric properties such as positions, orientations, and occupied spaces. We can use them as landmarks in SLAM. With the development of deep learning, the object detection algorithm [13] can provide bounding boxes with semantic labels representing object areas in images. It adapts well to viewing angle changes, illumination, and occlusion, and can serve as a sensor to detect objects.

Quadrics, as higher-dimensional representations than points, lines, and planes, have a well-grounded multiple view geometry theory [14] and can compactly model the position, orientation, and shape of the object. QuadricSLAM [15] first proposed introducing quadrics as an object model into SLAM and optimizing the quadrics parameters using the bounding boxes from the object detection in a factor graph. However, the monocular observation model makes the quadrics’ initialization difficult, as it needs to get at least three observations with massive viewing angle changes to converge. Since it is challenging to generate diversified viewing angles under a mobile robot’s planar motion model, unobservable problems [16] happen easily. Moreover, QuadricSLAM leaves the data association problem unsolved.

We propose to introduce depth data into the object-level SLAM system. An RGB-D camera is commonly used for mobile robots, which is low-cost and commercially available. It offers RGB images and depth data and is suitable for indoor environments. Laser and Lidar generate a 2D or 3D point cloud specifically. However, they lack visual information for scene detail. Combining a monocular camera with a Lidar may be a suitable solution for implementing our algorithm in outdoor environments. Currently, we will discuss the RGB-D camera only in this paper.

Based on previous research, we propose a sparse object-level SLAM using quadrics based on RGB-D cameras commonly used by mobile robots. We propose two types of RGB-D camera-ellipsoids observation models: complete observation model and partial observation model. The complete model extracts a complete ellipsoid from a single RGB-D observation based on the relationship between the object and its supporting plane. Aiming at the point cloud missing problem caused by occlusion and invalid depth data in the RGB-D frame, when the missing is severe, we use the bounding boxes from object detection to form a partial observation model. We propose a novel evaluation method to flexibly switch the two observation models to adapt to the received RGB-D data. For the data association problem that the state-of-the-art algorithm has not solved yet, we introduce a nonparametric pose graph [17] to solve in the back end robustly. The conventional SLAM tends to evaluate the trajectory quantitatively and ignores the map. Instead, we address the mapping evaluation and propose a benchmark that considers objects’ poses, shapes, and map completeness to comprehensively evaluate the mapping effect. We compared our algorithm with two state-of-the-art object-level algorithms, QuadricSLAM [15], based on a monocular quadric observation model, and a point-model object SLAM [17], based on a nonparametric pose graph using points as an object model. We ran experiments on two public datasets, ICL-NUIM [18] and TUM-RGB-D [19], and an author-collected mobile robot dataset in a home-like environment.

In summary, the contributions of our work are as follows:

- We propose an object-level SLAM algorithm using quadrics as an object model and propose two RGB-D quadric observation models.
- We propose a method to extract a complete ellipsoid from a single RGB-D frame based on the relationship between an object and its supporting plane.
- We innovatively introduce a nonparametric pose graph to the quadrics model to solve the semantic data associations in the back end.
- We have thoroughly evaluated the proposed algorithm’s effectiveness compared with two state-of-the-art object-level SLAM algorithms on two public datasets and an author-collected mobile robot dataset in a home-like environment.

## 2. Related Work

### 2.1. Representations in Visual SLAM

Visual SLAM systems observe landmarks from different poses and construct constraints to solve the camera poses and the landmarks’ locations [1]. Conventional visual SLAM systems generally use point features [8,9,20], line features [12], and plane features [10,11] as landmarks. Those low-dimensional geometric representations can help the robot locate its poses, while the lack of semantic information in the map limits the mobile robot’s ability to understand the environment. Thus, those representations cannot meet semantic navigation and advanced human–robot interaction.

Human beings understand the environment through geometrics and semantics of objects. The earliest work introducing objects as independent landmarks into SLAM, SLAM++ [21], establishes a database of computer aided design (CAD) models of objects in advance and uses depth data of RGBD camera to retrieve the object in the database during actual operation. The dependence of the database limits its ability and cannot detect objects outside the database. Later, MaskFusion [22] does not require a priori database. It uses Mask-RCNN [23] for instance-level object detection and segmentation and uses the Surfels model to store objects. Similarly, Fusion++ [24] and MID-Fusion [25] use the TSDF model and octree voxel model to store objects. Recently, in the field of semantic 3D mapping, Kimera [26] builds a 3D mesh of the environment with semantic labels. However, the object models mentioned above are all dense, and their goal is to finely construct the surface of objects, which brings a high amount of computing resources. Delicate geometric surfaces are useful in structure from motion and augmented reality (AR) applications. For mobile robots’ navigation tasks, more attention is paid to the position, orientation, and shape of objects.

Recently, Yang et al. proposed CubeSLAM [27], which uses a three-dimensional cuboid model to represent objects in the environment. It recovers the three-dimensional cuboid from monocular images using bounding boxes from object detection and vanishing points. However, the extraction of vanish points requires the objects to have clear parallel straight lines on the surface, which limits the applicable object categories.

### 2.2. Observation Models of Quadrics

The quadrics models [14], such as an ellipsoid, have well-grounded mathematics in multiple-view geometry. It can model the position, orientation, and occupied space of objects compactly. The earliest work [28,29,30] studied the structure from motion problems of recovering the quadric model based on the bounding boxes from object detection but limited to mapping and did not estimate the camera poses. QuadricSLAM [15] tried for the first time to introduce ellipsoids as object models into the graph optimization of SLAM and built a map while estimating the camera poses. After that, many works made improvements on its basis. Jablonsky et al. [31] explored the use of the gravity direction to constrain the ellipsoid. Ok et al. [16] introduced object size to improve mapping. Hosseinzadeh et al. [32] proposed a SLAM system with points, planes, and quadrics. They also introduced the constraint between the supporting plane and the ellipsoid into the graph optimization. Gaudilliere et al. [33] explored the relocalization problem based on the quadrics model. Successive works are proving that the quadrics model is feasible for modeling objects in environments, due to its compact representation and well-grounded geometric theory.

However, the object SLAM systems based on quadrics mentioned above are all based on monocular cameras and are limited by monocular cameras’ observation model. An ellipsoid’s recovery requires more than three observations of bounding boxes and requires a massive enough viewing angle change between each observation. A mobile robot usually moves in planar circular motion modes. The motion lacks viewing angle changes in the vertical and pitch directions, which easily causes unobservable problems [15]. To address the problem, Ok et al. [16] estimated a texture plane based on the feature points on the object surface and introduced an object size prior as new constraints. The algorithm requires the object’s surface to have apparent texture and known object size prior, which limits the application range. Hosseinzadeh et al. [34] proposed to use a CNN model to recover the three-dimensional object point cloud from a single-frame image. However, the proposed network model requires a large amount of training data, and the generalization ability in the real environment needs further verification.

Unlike the research mentioned above, this paper introduces the RGB-D camera’s depth data to help solve the unobservable problems. However, RGB-D cameras face problems such as a loss of point cloud data caused by occlusions, illuminations, and small fields of view. In the field of manipulators’ grasping, researchers used RGB-D cameras to recover the object as a quadric to calculate grasping points [35,36,37,38]. Schiebener et al. [35] proposed using supporting planes and symmetric properties of objects to complement the point cloud to address the occlusion problem. Vezzani et al. [36] and Makhal et al. [37] complemented the object’s point cloud by symmetric properties and then fitted the quadric models. Vezzani et al. [38] further introduced a priori information about the object’s shape during the quadric fitting.

To analyze the grasping point and better fit the object’s contour, the above work uses the higher-dimensional model SuperQuadrics. This paper uses the quadrics model to emphasize the compact expression of the object’s pose and shape to build an object-level semantic map. The above research only uses single-frame RGB-D observation. We introduce a graph optimization framework to integrate multiframe observations to estimate globally consistent ellipsoid parameters. The research background of the above work is small objects placed on the desktop. Ours is for mobile robot navigation, which emphasizes the diversity of the actual environment, adapting to small objects on the desktop and large objects such as furniture. Besides, this paper proposes a new object orientation estimation method using the supporting relationship and the normal vector histogram of the object surface to construct more constraints to improve the mapping’s accuracy and robustness.

### 2.3. Semantic Data Associations

Conventional visual SLAM uses the descriptor of point features [6,7], or the geometric difference of line and plane features [10,11,12], to track and solve data associations of observations between frames. After associations solved at the front end, it is fixed during the optimization in the back end. The observation of object-level SLAM comes from the bounding boxes output by the object detection algorithm. Compared with the manually designed features of conventional visual SLAM, deep learning algorithm training on large amounts of data can effectively adapt to massive view angle changes [13]. However, it poses challenges for data associations. First, compared with the point-features SLAM extracting hundreds of feature points in an image, object SLAM contains only several object observations. Wrong data associations will bring significant impacts to the system. Second, bounding boxes only include the regions and semantic labels in the image. However, there are many objects in the real cluster environment, and their distances are relatively close. There are multiple instances of objects with the same labels, such as books, cups, chairs. In the presence of observation errors, it is challenging to distinguish only by semantic labels and bounding box. Third, limited by the viewing angle, only a part of the object can be seen when the occlusion exists.

Iqbal et al. [39] proposed using the distribution of point clouds back-projected from bounding boxes as features and solving the association in the front end. However, under partial occlusion, the distribution of object point cloud is significantly different from the complete one. To explore a more robust association, the researchers proposed solving the data association in the back end and putting it as an unknown variable into the graph optimization to obtain a globally consistent solution. As the data association variables are discrete, camera and landmarks poses variables are continuous, the system becomes a non-Gaussian hybrid model [17,40,41]. Bowman et al. [40] derived probabilistic data associations and integrated them with SLAM and use an expectation-maximization (EM) solution to marginalize data associations to maintain Gaussian distribution. However, traversing all the associations of the previous poses brings a tremendous amount of computation. Doherty et al. [41] introduced a nonparametric inference in the factor graph and proposed a confidence propagation method to solve it, but it required a hard decision to add new objects. Mu et al. [17] proposed introducing a nonparametric pose graph for object-level SLAM and used Dirichlet Process to model the process, which can adapt to the situation where the number of objects in the map is unknown.

Aiming at the unresolved data association problem in the state-of-the-art quadric SLAM, we innovatively propose to combine the observation model of the quadrics with the nonparametric pose graph to solve the data association robustly in the back end. It is worth mentioning that other semantic association methods mentioned above can also use the quadrics observation model proposed in this paper. The study of the effectiveness of various association methods with the quadrics model is also one of our future research directions.

## 3. Materials and Methods

### 3.1. System Overview

**Figure 1:**
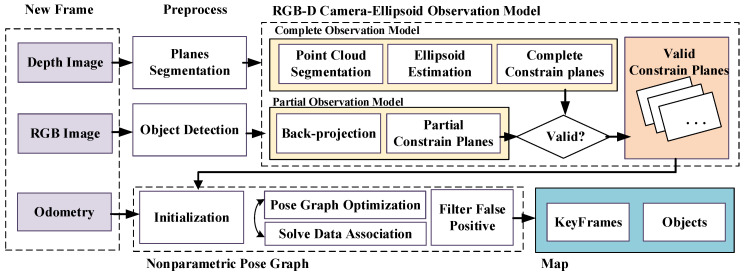
The algorithm flow chart.

Figure 1 shows the complete framework of the proposed algorithm in this paper. A new frame composed of RGB-D images and odometry data goes through the preprocess modules and the RGB-D camera-ellipsoid observation model to generate valid constraint planes. All the observations will then participate in the nonparametric pose graph to get globally consistent solutions, which contain poses of keyframes and the parameters of objects. First, we will introduce the RGB-D camera-ellipsoid observation model in detail.

### 3.2. Quadrics Model

There are many types of quadrics, such as ellipsoids, hyperboloids, cylinders. Considering that indoor artificial objects generally have a closed geometric shape, this paper uses the ellipsoid in the quadrics family as the object model. Hartley et al. [14] discussed the mathematics model and projection geometry of the quadrics in detail. This part briefly summarizes to help to understand the paper.

A quadric surface can be expressed by a dual form $Q^{*}$. For any plane π tangent to it, the relationship can be expressed compactly as a plane constraint formula:

(1) $$\pi^{T}Q^{*}\pi \;=\;0.$$

**Figure 2:**
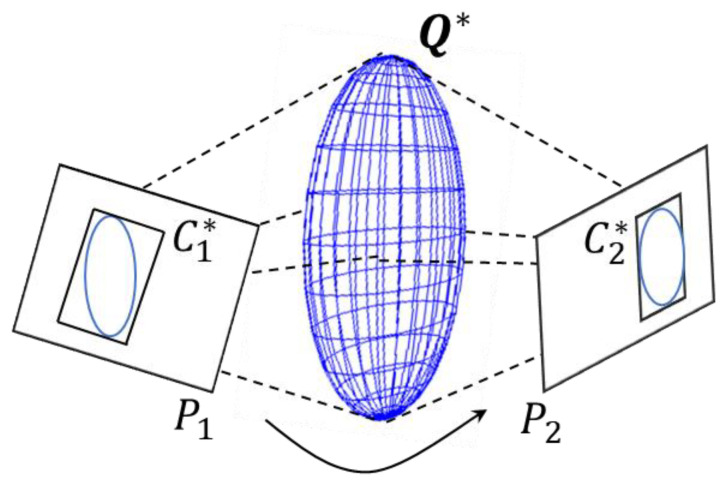
The camera projection model of the ellipsoid. An ellipsoid is expressed in the dual form $Q^{*}$. Under the observation of different camera poses P, different ellipses $C^{*}$ are projected on the image plane. The projected ellipses can establish constraints with the bounding boxes from object detection.

In the RGB-D observation model described later, we will use the plane constraint formula to form the constraint on the ellipsoid. The dual quadric surface $Q^{*}$ is projected under the camera observation P to obtain the curve $C^{*}$, as in Figure 2. This process can be conveniently expressed as:

(2) $$C^{*}=PQ^{*}P^{T},$$

where P=K[R|t], K is the camera calibration matrix, R, t are the camera rotation and translation. When the quadric surface is an ellipsoid, the projection curve $C^{*}$ is an ellipse. In the complete constraint model described later, we will evaluate the confidence of the estimated ellipsoid through the projection.

### 3.3. RGB-D Camera-Ellipsoid Observation Model

The state-of-art SLAM algorithms based on the quadric model, such as QuadricSLAM [15], use the projected ellipse from the ellipsoid in the map (Equation (2)) to construct a constraint with the bounding box from object detection. As one bounding box can only constrain four degrees of freedom and an ellipsoid has nine degrees of freedom, they require at least three observations to completely constrain an ellipsoid. Also, those observations need to ensure massive viewing angle changes between each other. Considering the moving modes of mobile robots, the viewing angle changes in the vertical and pitch directions are limited, which easily causes unobservable problems [15,16].

To reduce the monocular observation model’s requirements for the number of observations and viewing angle changes, we introduce the depth data of the RGB-D camera commonly used in mobile robots. We extract the object supporting plane from the depth data and introduce the object-plane support relationship to help segment the object point cloud and estimate the object orientation. To reduce the requirement for viewing angle changes, we derive the RGB-D complete observation model and use a single frame of RGB-D data to estimate complete ellipsoids to achieve fast initialization. To deal with the severe missing of depth data caused by occlusion or illumination, we propose a partial constraint model using bounding boxes from object detection to construct constraints to maximize observation information.

**Figure 3:**
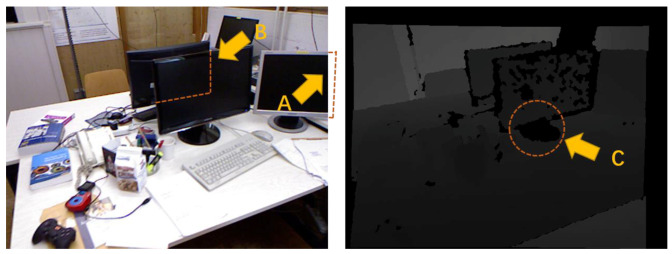
Classic challenges of RGB-D observations. (**A**) Occlusions by image edges; (**B**) occlusions by objects; (**C**) invalid depth values.

Although RGB-D observation provides depth information, the following problems still need to be solved in actual use, as shown in Figure 3: (1) occlusions caused by the edge of the image; (2) occlusions of other objects in a cluttered scene or the object itself; (3) the depth data affected by illumination and material reflection, which easily causes voids. The defects mentioned above will cause the incomplete point cloud of the object and pose a challenge to the observation model.

**Figure 4:**
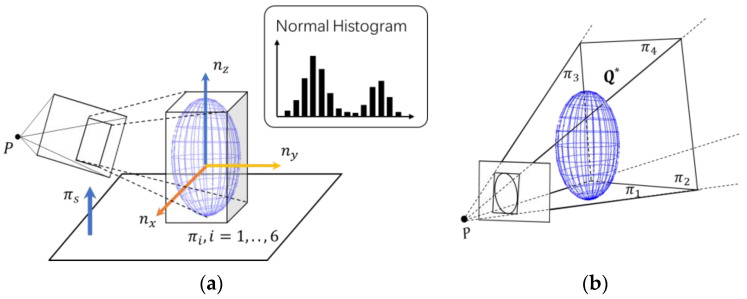
Two types of RGB-D camera-ellipsoid observation models proposed in this paper. (**a**) Complete observation model; (**b**) partial observation model. The complete observation model estimates a complete ellipsoid and uses the tangent planes of its circumscribed rectangle as constraints. The partial observation model is activated when the point cloud information is insufficient and uses the frustum planes generated by the bounding box to constrain the ellipsoid.

According to the completeness of point cloud data, this paper proposes two observation models: a partial constraint model and a complete constraint model, as shown in Figure 4. When the point cloud quality is high, the algorithm applies the complete constraint model to recover a complete ellipsoid from a single frame of RGB-D data. This model makes reasonable use of the support relationship between indoor objects and space planes and estimates the object’s pose and shape. Finally, the algorithm establishes a circumscribing cuboid with six planes of the estimated ellipsoid. These planes form a tangent relationship with the ellipsoid, and the plane direction is orthogonal to the principal axis of the ellipsoid. In this way, we obtain a complete constraint model with up to nine degrees of freedom.

If there is a significant occlusion or the point cloud quality is challenging to estimate a complete ellipsoid, the algorithm will enable the partial constraint model. This model makes full use of the constrained planes generated by the bounding box from object detection and can produce constraints with up to four plane tangent relations.

We propose a novel evaluation function to judge the estimated ellipsoid quality to realize the flexible switching of the two types of observation models. We apply the proposed observation models and the evaluation function to the following aspects of the object-level semantic SLAM framework:

- We use the estimated ellipsoids in the complete constraint model to initialize new objects;
- We use the constraints models as cost functions in the graph optimization and solve data associations.

### 3.4. Complete Constraint Model

**Figure 5:**
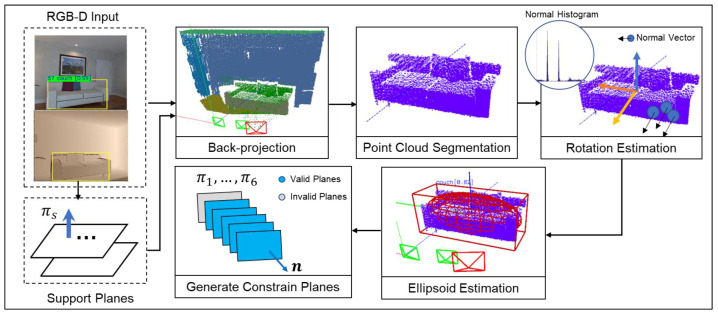
The process of extracting a complete ellipsoid from a single RGB-D frame.

The complete constraint model makes full use of artificial objects’ properties and their relations with structure planes. It tries to recover the complete ellipsoid parameters from one frame of RGB-D observation based on those properties. Figure 5 shows the process.

For the estimated ellipsoid, we propose an evaluation method to measure its effective probability and keep it if it meets the threshold. Otherwise, the algorithm will switch to the partial observation model to maintain maximum information utilization and maintain its constraint confidence.

#### 3.4.1. Supporting Planes Segmentation

**Figure 6:**
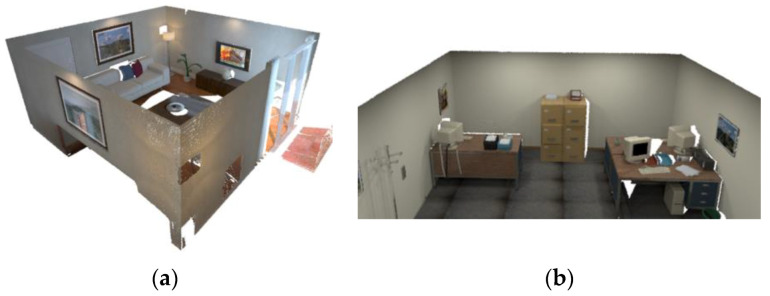
Typical scenarios with the Manhattan assumption. (**a**) Living room; (**b**) office.

Artificial environments, such as homes, offices, and other settings, often satisfy the Manhattan world assumption [42], that is, they have large-scale planar structures such as a ground plane, walls, and desktops that keep orthogonal or parallel to each other, as shown in Figure 6. Artificial objects in indoor environments have some ubiquitous properties. If not suspended, they must have a supporting plane to balance their gravity.

Reasonable use of those properties can help us segment the point cloud and provide a priori constraints on the ellipsoid rotation. To avoid repetition, this section takes the most common indoor support relationship as an example to derive the ellipsoid extraction process. We can obtain the suspension relationship of the roof and the wall similarly.

First, based on our previous work [10], we extract planes using Random Sample Consensus (RANSAC) in the depth image, and record them as a plane set $S_{0}=\left\{\pi_{i}\right\}$. We make a reasonable assumption that the supporting planes of objects are vertical to the gravity direction, such as a ground plane, desktops. Then, we estimate a rough gravity direction $n_{g}$ to select potential supporting planes from all the planes in the image.

For most service robots in indoor environments, their supporting planes are the ground plane. Generally, the installation pose of the RGB-D camera on the robots is set in the factory and fixed during the operation with little vibration. We can get an estimation of the gravity direction from the transformation matrix of the camera to the robot body. For hand-held situation, if an Inertial Measurement Unit (IMU) is available beside the RGB-D camera, the gravity direction could be estimated from the IMU more flexibly to the environments. Otherwise, there is also a proposed robust method to estimate the ground plane [43].

We get the gravity direction $n_{g}$ from the ground plane. For each plane $\pi_{i}\in S_{0}$, if the angle between its normal and the gravity direction is less than $\varepsilon_{0}$, we select it as a potential supporting plane. We put all the potential supporting planes to a plane set $S=\left\{\pi_{i}\right\}$. We use $\varepsilon_{0}=10$ degrees in the experiments.

#### 3.4.2. Object Point Cloud Segmentation

As in Figure 5, according to the bounding box $b_{i}$ from the object detection algorithm, we can obtain the object’s rough point cloud by back-projection from the depth image. Due to the observation angle limitation, the rough point cloud often contains part of the supporting plane or other objects. We introduce a lightweight segmentation method to segment the point cloud belonging to the object simply and efficiently to keep the real-time performance. We filter the points belonging to the supporting plane and then keep the points $C_{i}$ belonging to the object by Euclidean filtering.

Then, for a given frame of RGB-D data, the bounding box of each object in the image and the corresponding object envelope point cloud $\left\{b_{i},C_{i}\right\}$ are obtained. Later, we will design a probability to evaluate segmentation success. Although the ellipsoid is a rough envelope of the object contour, the fusion of multiframe observations in SLAM is beneficial to improve the robustness and obtain a globally consistent map.

#### 3.4.3. Object Orientation Estimation

As RGB-D data is limited by occlusion or lack of depth data, the point cloud is often incomplete, and it is challenging to estimate its orientation accurately. We propose an object orientation estimation method based on the relationship between indoor artificial objects and the supporting planes. This method is based on two reasonable assumptions: (1) One of the object’s main axes is parallel to its supporting plane’s normal vector. (2) For artificial objects with dominant orientation directions, the histogram of normal vectors on the object’s surface can reflect the direction trend. We will elaborate on it as below.

(A) Definition of object coordinate system

We define the object coordinate as in Figure 4a. When the object is placed on its supporting plane, the *Z*-axis represents its upward coordinate axis along the direction of gravity, and the *Y*-axis and the *X*-axis represent the orthogonal dominant directions on the surface of the object. As an ellipsoid is symmetric, if we rotate it 90 degrees around its *Z*-axis four times, we can get four rotation matrices representing the same rotation of the ellipsoid. We use the dominant direction of an object in its supporting plane as its *Y*-axis for convenient derivation. Later, we will show how to represent the complete constraints by planes to uncouple from the axes.

First, we estimate the *Z*-axis $n_{z}$ of the object and one of the dominant directions *Y*-axis $n_{y}$. We can obtain the remaining *X*-axis $n_{x}$ according to the orthogonal property of the right-handed system:

(3) $$n_{x}=n_{z}\times n_{y}.$$

Then the rotation matrix R of the object is composed of the three vectors:

(4) $$R=\left[n_{x},n_{y},n_{z}\right].$$

(B) *Z*-axis estimation based on the supporting plane

Indoor objects are mostly located on a supporting plane. Large furniture, such as chairs, sofas, cabinets, are located on ground planes, while small objects such as water cups, mice, keyboards, are located on desktops. In these cases, an object and its corresponding supporting plane form a vertical relationship, so the *Z*-axis of the object is parallel to its supporting plane’s normal vector. In the process of point cloud segmentation, we have obtained the supporting plane $\pi_{s}$ of the object. Assuming its normal vector is $n_{s}$, the *Z*-axis $n_{z}$ of the object is:

(5) $$n_{z}=n_{s}.$$

(c) *Y*-axis estimation based on the histogram of normal vectors

**Figure 7:**
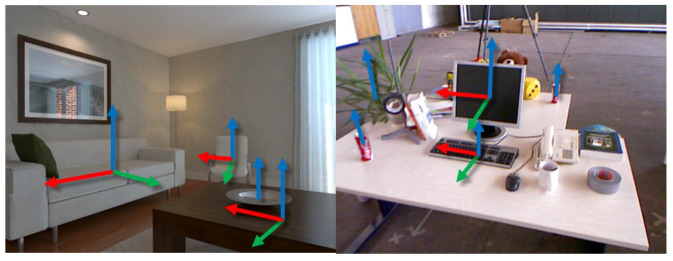
We show examples of objects with a dominant direction, marked with a complete coordinate, and objects without dominant direction, marked with *Z*-axis only.

As in Figure 7, the surface of most artificial objects has orthogonal dominant directions when projected to its supporting plane, such as monitors, sofas, chairs. We can use their rotations as constraints. While some objects with rotational symmetry do not, such as objects with the shape of spheres and cylinders like bottles, dishes, or objects with no specified shape like potted plants. Considering the orientation for those objects is meaningless. We propose introducing the semantic label of the object to choose whether to activate the dominant direction constraints of the object. Humans apply many a prioris about objects’ semantics when perceiving objects’ shapes, such as symmetry and recurrence. Here we will demonstrate how object semantics can help and participate in the constraints of SLAM.

First, we build an a priori table of the dominant direction’s validity in its supporting plane according to the semantic label of the object. We activate the orientation constraints for the *X*-axis and *Y*-axis in graph optimization only for the type of objects with valid dominant directions. We offer a simple way to judge its validation using object labels and an a priori table. It could be a valuable future work for how to obtain such an a priori table robustly and automatically. For example, analyzing the object’s geometric surface and finding out whether the orientation data of multiple observations have a gathering tendency could determine whether to activate the orientation constraint.

Regardless of whether the object’s rotation angle is valid in the graph optimization, to obtain a complete ellipsoid, we will first estimate the *Y*-axis for all objects according to the following algorithm.

To better represent *Y*-axis $n_{y}$, we establish a polar coordinate system on the supporting plane $\pi_{s}$. We take a unit vector $n_{r}$ in the plane as the coordinate axis. For any vector n in the supporting plane, we define yaw angle $\theta =\mathrm{acos}\left(n\cdot n_{r}\right)$ as the angle between $n_{r}$ and n in the polar coordinate.

For artificial objects with dominant directions in the supporting plane, the histogram of the yaw angles of the object point cloud’s normal vectors has a peak in the dominant direction, and we choose the peak as the yaw angle of its *Y*-axis. The algorithm shows in detail as follows:

1. For all points $P\in C_{i}$ in the object point cloud, complete steps 2–4 to calculate the yaw angle by projecting its normal vector on the supporting plane.
2. Calculate its normal vector $n_{p}$ according to its local depth information. Since the normal vector of the entire depth map has been calculated when extracting the Manhattan plane, it can be retrieved directly.
3. Project the normal vector $n_{p}$ into the supporting plane $\pi_{s}$ to obtain the yaw angle θ. Suppose the normal vector of the supporting plane $\pi_{s}$ is $n_{s}$, first find the component $n_{\mathrm{ps}}$ of the normal vector $n_{p}$ in the supporting plane $\pi_{s}$:
  (6) $$n_{\mathrm{ps}}=n_{p}-\left(n_{p}\cdot n_{s}\right)n_{s}.$$
  The normal vectors on the right are all unit vectors, let $n_{\mathrm{ps}}=\frac{n_{\mathrm{ps}}}{\left|n_{\mathrm{ps}}\right|}$ to normalize it.
4. Calculate yaw angle $\theta =\mathrm{acos}\left(n_{\mathrm{ps}}\cdot n_{r}\right)$. Normalize θ to [0,π), add θ to the set Y.
5. Draw a histogram of all angles in Y with the parameter angle ω as the interval (we use ω=5 degrees in the experiments). Suppose interval C includes all angles in the angle range $\left[C-\frac{\omega }{2},C+\frac{\omega }{2}\right)$, and let function num(C) be the number of normal vectors counted in interval C. According to the assumption that indoor objects have dominant directions, we can find the peak by the following formula:
  (7) $$C^{*}=\underset{C}{\max}\;\left(\mathrm{num}\left(C\right)\right),\;C\in \left[0,\pi \right).$$
6. Take the median value of the interval $C^{*}$ for the yaw angle θ in the dominant direction and calculate the corresponding normal vector form $n_{x}$. It rotates the polar coordinate axis $n_{r}$ by the angle θ around the normal vector $n_{s}$ of $\pi_{s}$. First, obtain the rotation matrix $R_{\theta }$ of the process by Rodriguez’s formula:
  (8) Rθ=cos(θ)I+(1−cos(θ))nsnsT+sin(θ)ns^,
  where ns^ represents the antisymmetric matrix of the vector $n_{s}$. Then get the normal vector $n_{x}$:
  (9) $$n_{x}=R_{\theta }n_{r}.$$

Finally, we give the process a confidence probability $P_{\mathrm{rot}}$ based on its dominance. We believe that the more the normal vector is concentrated on the peak, the more the dominance. The total number of normal vectors is $n_{\mathrm{all}}$, and we count the proportion of the number in the peak interval to the whole as the dominance probability:

(10) $$P_{rot}=\frac{\mathrm{num}\left(C^{*}\right)}{n_{\mathrm{all}}}.$$

#### 3.4.4. Ellipsoid Generation

In the above steps, we obtained the object’s rotation matrix $R_{c}$. Based on the object point cloud and its rotation matrix, we find the point cloud’s minimum bounding cuboid. Find the semiaxis length of the point cloud along the three main axis directions as $s={\left[a,b,c\right]}^{T}$ of the cuboid and take the center of each axis as the cuboid center $t_{c}$. Then, the pose of the cuboid is $T_{c}=\left[\begin{array}{cc} R_{c} & t_{c}\\ 0 & 1 \end{array}\right]$.

For any cuboid, there is a unique inscribed ellipsoid. We propose to define the inscribed ellipsoid of the cuboid C as the ellipsoid model Q of the object. Let D=diag(s) be the diagonal matrix generated by the vector s, we obtain the dual parameter $Q^{*}$ as below:

(11) $$Q^{*}=T_{c}\left[\begin{array}{cc} DD^{T} & 0\\ 0 & -1 \end{array}\right]T_{c}^{T}.$$

So far, we extract the ellipsoid from a single frame of RGB-D. Compared with the cuboid form, the ellipsoid model has a more unified mathematical expression. To consider the occlusion of the real scene to improve the robustness, while conveniently participating in the solution of graph optimization and data association, we continue to derive an error function constrained by the tangent planes. The tangency constraint between the surface and the ellipsoid can be expressed compactly by the formula, and later we will prove that the constraints between the bounding boxes and the ellipsoid in the partial observation model can also be converted into plane constraints, realizing the unification of the complete and partial constraints.

#### 3.4.5. Confidence Evaluation of the Complete Constraint Model

Since the bounding boxes from object detection are reliable object constraints, we propose to measure the geometric relationship of the ellipsoid estimated in the complete constraint model and the bounding box as an indicator of the complete constraint model’s confidence.

First, we project the ellipsoid into the image plane to obtain an ellipse $c^{*}$ and define the ellipsoid shape matching probability $P_{\mathrm{shape}}$ as the IoU (Intersection over Union) of the circumscribed rectangle $\beta \left(C^{*}\right)$ of C and the bounding box $b_{i}$. If the camera projection matrix is P, according to formula (2), the matching probability of the ellipsoid shape is:

(12) $$P_{\mathrm{shape}}=\mathrm{IoU}\left(\beta \left(C^{*}\right),b_{i}\right)=\mathrm{IoU}\left(\beta \left(PQ^{*}P^{T}\right),b_{i}\right).$$

The shape matching probability can evaluate the point cloud segmentation error and the missing caused by occlusions or invalid depth data. When the extraction process is successful, the ellipsoid should be as close to the bounding box as possible to produce a higher IoU.

We form the ellipsoid’s complete confidence by combining the probabilities of each step in the ellipsoid extraction process according to the Bayesian formula, including the object detection probability $P_{\det}$, the dominant direction estimation probability $P_{\mathrm{rot}}$, and the shape matching probability $P_{\mathrm{shape}}$. To finally determine the availability of the complete ellipsoid constraint model, the following evaluation function is defined:

(13) $$P_{e}=P_{\det}P_{\mathrm{rot}}P_{\mathrm{shape}}<\alpha ,$$

where α is the confidence threshold (We use α=0.1 in the experiments). When the condition (13) meets, we consider that the information in the RGB-D observation of the current frame is not enough to generate a reliable ellipsoid, and we will switch to the partial constraint model to ensure the reliability of the constraint. Otherwise, we consider this ellipsoid reliable and continue to generate constraint planes.

#### 3.4.6. Generate Constraint Planes

(A) Acquisition of tangent plane

When the occlusion exists, the estimated ellipsoid may only be a part of the object rather than the whole. To flexibly adapt to occlusion, we innovatively propose to split the constraint of an ellipsoid into multiple discrete constraints. Remember that its circumscribed cuboid contains six orthogonal planes $S_{c}$ for an ellipsoid, for each plane $\pi ⋴S_{c}$, it satisfies the tangency constraint of the formula (1) with the ellipsoid $Q^{*}$. We propose to represent the complete constraint using those constraint planes.

The complete observation model also includes orientation constraints, which contain two types. One is the *Z*-axis direction constraint brought by the supporting plane, universal to all objects on the plane. The other type is only valid for objects with a dominant direction on the surface, which are the valid objects in Table 1. We represent the angle constraint using its corresponding constraint plane. Specifically, for a plane of the ellipsoid’s circumscribed rectangle, the minimum angle difference between the normal vector of the plane and the three principal axes of the ellipsoid should be 0. Given the rotation matrix of an ellipsoid $R=\left[n_{\mathrm{qx}},n_{\mathrm{qy}},n_{\mathrm{qz}}\right]$, for a tangent plane π orthogonal to the ellipsoid, let the unit vector $n_{p}$ be its normal vector, and it must be collinear with one of the axes of the ellipsoid, which is:

(14) $${||R^{T}n_{p}||}_{1}-1=0,$$

where ${||a||}_{1}=\sum \left|a_{i}\right|$, is the 1-norm of the vector a. When the orthogonality condition is met, $n_{p}$ is parallel to one of $n_{\mathrm{qx}},n_{\mathrm{qy}},n_{\mathrm{qz}}$ and perpendicular to the other two axes. Assuming it is parallel to $n_{\mathrm{qx}}$, the three elements’ absolute values of the vector $R^{T}n_{p}$ are ${\left[1,0,0\right]}^{T}$, so that the 1-norm is 1. When the condition is not met, the 1-norm will be larger than 1.

Therefore, a complete constraint plane contains two parts of constraints on the ellipsoid Q: tangent constraint and angle constraint. Suppose the covariance matrix of the tangent constraint is $\Sigma_{d}$, and the covariance matrix of the angle constraint is $\Sigma_{\theta }$. Assuming that the object L contains the ellipsoid parameter $Q^{*}$ and the semantic label l of the object, R is the rotation axis of the ellipsoid $Q^{*}$, and $n_{p}$ is the normal vector of the plane π, then the overall constraint function is:

(15) $$f_{g}\left(\pi ,L\right)=||\pi^{T}Q^{*}\pi {||}_{\Sigma_{d}}+Sem\left(\pi ,l\right)||||R^{T}n_{p}||{}_{1}-1{||}_{\Sigma_{\theta }},$$

where, Sem(π,l) is a function for judging whether the angle constraint of the plane π is valid,

(16) $$Sem\left(\pi ,l\right)=\{\begin{array}{cc} 1 & \left(l\in U_{\mathrm{valid}}\right)\;OR\;\left(\pi \in \Pi_{Z}\right),\\ 0 & \mathrm{otherwise}, \end{array}$$

where, $\Pi_{Z}$ is a set of planes perpendicular to the Z-axis, and $U_{\mathrm{valid}}$ is the object category with valid dominant directions in Table 1. In this way, the semantic categories of objects participate in the construction of geometric constraints.

Finally, the complete constraints are composed of six constraint planes and their angle constraints with the ellipsoid. Next, we can judge the validity of the plane to deal with the occlusion.

(B) Occlusion of the image edges

When the bounding box is located at the edge of the image, the estimated complete ellipsoid is only a part of the actual object. As we express the constraints in the form of tangent planes, we can determine each tangent planes’ effectiveness, remove invalid constraints caused by occlusion, and retain valid constraints.

Since the tangent planes come from the ellipsoid’s circumscribed rectangle, each tangent plane is composed of four vertices of the rectangle. We propose to decide whether a tangent plane is valid by judging the number of its projected vertices that locate within the image boundary. The specific process is as follows:

1. For a tangent plane π, project the cuboid’s four corresponding vertices into the image plane to obtain the projection point $\left\{u_{i}\right\}$.
2. Check whether projection point $u\in \left\{u_{i}\right\}$ is within the image, that is, its distance to the image boundary is larger than the threshold ρ, and count the number of points within the image as $n_{b}$. We use ρ=10 pixel in the experiments.
3. Set the filter parameter $n_{t}$, if $n_{b}<n_{t}$, mark the plane as an invalid plane. We use $n_{t}=3$ in the experiments.

After filtering out the invalid planes, we put valid planes constitute to a set $\bar{S_{c}}$. Therefore, the final complete constraint equation is:

(17) $$F_{g}\left(\bar{S_{c}},L\right)=\Sigma_{\pi }f_{g}\left(\pi ,L\right),\;\pi \in \bar{S_{c}}.$$

### 3.5. Partial Constraint Model

When the point cloud is seriously missing due to severe occlusion of the viewing angle or illuminations, it is challenging to obtain a complete ellipsoid. In this case, the final probability $P_{e}$ of the complete constraint is low, and the algorithm will switch to the partial constraint model. The partial constraint model will use the bounding boxes from object detection to construct constraints, as shown in Figure 4b. We propose to use the constraint planes to represent both the complete model and the partial model, expecting to obtain better convergence performance during graph optimization.

(A) Acquisition of constraint planes

The object detection algorithm generates a set of bounding boxes $\left\{b_{i}\right\}$ from the RGB image, where $b=\left(x_{\min},y_{\min},x_{\max},y_{\max}\right)$. Assuming the camera’s internal parameter matrix K, and the camera’s pose R, t, we can obtain the camera matrix P=K[R|t]. Therefore, each line of the bounding box can form a plane by back-projecting from the camera center. If the line is l, the generated plane π can be obtained by:

(18) $$\pi =P^{T}l.$$

The bounding box’s four edges can form a total of four constraint planes to form a set $S_{p}$.

(B) Occlusion of the image edges

Like the complete constraint model, we introduce an occlusion situation to the partial constraint model. Due to the occlusion of the image’s edge, the edges of some bounding boxes are not directly tangent to the object. We follow the same threshold ρ defined in the complete constrain model to filter the bounding boxes’ occluded edges. Similarly, when the distance of an edge of the bounding box is less than ρ to the edge of the image, the edge will be ignored. Therefore, for the remaining edges of the bounding boxes that meet the conditions, the valid constraint planes can be generated by the formula (18), and a tangent plane cluster $\bar{S_{p}}$ can be formed. Assuming L contains the ellipsoid parameter $Q^{*}$ and the object label l, a tangent constraint of plane π to L is:

(19) $$f_{p}\left(\pi ,L\right)=||\pi^{T}Q^{*}\pi {||}_{\Sigma_{d}},$$

where the constraint variance is $\Sigma_{d}$. Then the partial constraint function is:

(20) $$F_{p}\left(\bar{S_{p}},L\right)=\Sigma_{\pi }\;f_{p}\left(\pi ,L\right),\;\pi \in \bar{S_{p}}.$$

When all the constraint planes are valid, the partial constraint contains four degrees of freedom. The partial constraint in formula (19) is the same as the complete constraint in formula (15) when the angle constraint is invalid. In this way, we have unified the observation constraints of two constrain models by constraint planes.

### 3.6. Object-Level SLAM

Section 4 derives the RGB-D camera-ellipsoid observation model. In this section, we introduce the quadrics model as landmarks into the back end. We also introduce the unknown semantic data association and the objects’ semantic labels as discrete random variables into the systems. As the entire system has become a mixed system containing continuous (camera poses, quadrics parameters) and discrete (data associations, semantic labels) variables, making the traditional nonlinear optimization solution of the pose graph invalid. We will show how to model the problem as a nonparametric pose graph and introduce a method to get the optimal solution.

#### 3.6.1. Semantic Data Association

Compared with conventional SLAM systems, the data associations for semantic SLAM face more challenges in the following ways. First, the conventional point-features SLAM system has hundreds of features in one image, while semantic SLAM only has several bounding boxes in one frame. Wrong data associations will bring a significant impact on the system. Second, object detection outputs a bounding box and a semantic label, however, there are often multiple instances of the same category of objects in the map. In cluster environments, objects are close and even in contact with each other. Therefore, it is challenging to match the observations between frames in the front end directly.

Mu et al. [17] introduced a nonparametric pose graph into object SLAM to solve the data association for the first time. It iteratively fixes and optimizes the data associations and other variables in the graph, such as camera poses and landmarks. It uses the point model for the object. We innovatively introduce a nonparametric pose graph with the quadric surface model to solve the data association problem. Compared with the point-model in [17], the quadrics models more information of objects, including orientation and occupied space in addition to the center. This section will first review the pose graph optimization problem, assuming the solved data associations, and then expand it to a nonparametric pose graph, where the data association is unknown.

#### 3.6.2. Pose Graph Optimization

**Figure 8:**
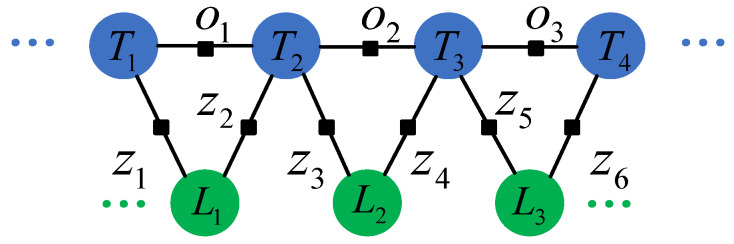
A factor graph for object simultaneous localization and mapping (SLAM). The node consists of the robot state T and the object landmark L expressed by the quadrics model. The edges are composed of camera-objects constraints z and odometry constraints o.

When the data association has solved, the back end of SLAM can be easily modeled as a factor graph with constraints between the odometry, camera poses, and landmarks, as shown in Figure 8. Assuming that the odometry is $o_{1:T}$, the sensor observation is $z_{0:T}$, the camera pose is $X_{0:T}$, the landmarks in the map are $L_{0:M}$, we can model SLAM problem as a maximum likelihood estimation [1]:

(21) $$\underset{X_{0:T},L_{0:M}}{\max}\log p\left(o_{1:T},z_{0:T};X_{0:T},L_{0:M}\right).$$

The maximum likelihood estimation problem can be modeled as the following nonlinear optimization problem:

(22) $$\hat{X},\hat{L}=\underset{X,L}{\arg\;\min}\left(\sum H\left(F_{z}\right)+\sum H\left(F_{o}\right)\right),$$

where $F_{z}$ is the camera-object observation constraint, and $F_{o}$ is the odometry constraint. H(·) is a robust kernel function. This paper chooses Huber Kernel to enhance the robustness of outliers.

#### 3.6.3. Camera-Object Observation Constraints

According to the RGB-D camera-quadric observation model derived above, there are two different types of camera-object observation constraints $F_{z}$, full constraint model $F_{\mathrm{full}}$ and partial constraint model $F_{\mathrm{part}}$, which are:

(23) $$\sum H\left(F_{z}\right)=\sum H\left(F_{\mathrm{full}}\right)+\sum H\left(F_{\mathrm{part}}\right).$$

For an RGB-D frame, the complete constraint model or the partial constraint model derived in Section 4 is represented by a set of tangent planes $\bar{S_{c}}$ in the camera’s coordinate system. According to the camera pose $H_{\mathrm{wc}}$ of the current frame, the tangent plane can be transformed into the world coordinate system $\bar{S_{w}}$:

(24) $$\bar{S_{w}}=\{\pi \;|\;\pi =H_{\mathrm{wc}}^{T}\pi_{c},\;\pi_{c}\in \bar{S_{c}}\},$$

where $H_{\mathrm{wc}}$ is the transformation matrix from the camera system to the world system corresponding to the current frame pose.

According to the known data associations, we find the corresponding object $L_{i}=\left\{Q_{i}^{*},l_{i}\right\}$ in the map, where its ellipsoid parameter is $Q_{i}^{*}$ and the semantic label is $l_{i}$. In this section, $l_{i}$ is a constant value and only $Q_{i}^{*}$ participates in the optimization. The camera-object observation constraint is the constraint of the plane cluster $\bar{S_{w}}$ on $Q_{i}^{*}$. According to whether the constraint is a complete constraint or a partial constraint, we activate different constraint functions. The constraint function for the complete constraint model is defined as follows:

(25) $$F_{\mathrm{full}}=F_{g}\left(\bar{S_{w}},L_{i}\right),$$

where $F_{g}$ is defined in Equation (17). Similarly, for partial observation constraints, the constraints are as follows:

(26) $$F_{\mathrm{part}}=F_{p}\left(\bar{S_{w}},L_{i}\right),$$

where $F_{p}$ is defined in Equation (20).

#### 3.6.4. Odometry Constraints

The input $o_{j}$ of the odometry constraint can be the wheel odometry of the mobile robot or the pose estimation result of the visual odometry algorithm, such as ORB-SLAM2 [20]. Defining the pose m(x,o) as the result of the pose x after a movement u, we can express the odometry constraint as:

(27) $$f_{o}\left(x_{j},o_{j},x_{j+1}\right)=||m\left(x_{j},o_{j}\right)⊖x_{j+1}||{}_{\Sigma_{j}}^{2},$$

where $\Sigma_{j}$ is the covariance matrix of the odometry, and ⊖ is an operator returns the relative pose in SE(3).

### 3.7. Nonparametric Pose Graph

Section 3.6 introduced the pose graph optimization assuming the data association is known. This section will introduce data associations as unknown variables. Besides, due to the uncertainty of object detection’s semantic label, we model it as a probability model and introduce it into the optimization in the back end. Mu et al. [17] proposed a method based on a nonparametric pose graph to solve the data association in the back end, using points as an object model. Instead, we propose to introduce the nonparametric pose graph with the quadrics model.

#### 3.7.1. The Definition of a Nonparametric Pose Graph

After introducing unknown data associations and semantic labels, we expand the pose graph introduced in formula (21) to the following joint optimization problem:

(28) $$\underset{X_{0:T},L_{0:M},y_{0:T}}{max}\log p\left(o_{1:T},z_{0:T},u_{0:T};X_{0:T},L_{0:M},y_{0:T}\right),$$

where $u_{0:T}$ is the observations of semantic labels, $y_{0:T}$ is the data associations, $L_{i}=\left\{Q_{i}^{*},l_{i}\right\}$ is the objects in the map, containing ellipsoid parameter $Q_{i}^{*}$ and semantic label $l_{i}$. The formula becomes a mixed-integer nonlinear problem. The number M of real objects in the environment is unknown and needs to be derived. Those differences bring many challenges to the solution of the formula.

#### 3.7.2. The Solution of a Nonparametric Pose Graph

**Figure 9:**
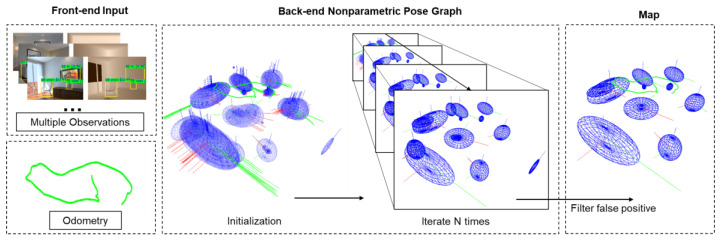
The solution process of the nonparametric pose graph.

Mu et al. [17] introduce Dirichlet Process (DP) means to solve the problem. The algorithm in [17] models objects as points. We introduce the proposed RGB-D quadric observation model with it. The DP means the algorithm is generally divided into two steps: fixing the data association y, maximize the likelihood to solve the variables X, L, and then fixing the variables X, L to solve the data association y according to the maximum likelihood. The iterative process is repeated until convergence. The process is shown in Figure 9.

In detail, it is divided into the following steps:

1. Initialization: According to the odometry data $o_{1:T}$, initialize poses X by an open loop. Initialize all objects L by considering all observations to be new objects and initialize the object label distribution β to the initial value $\beta_{0}$. Then execute steps 2–4 repeatedly until the algorithm converges.
2. Optimize data association: fix X, L, β, and update the posterior of each data association $y_{t}^{k}$ by the following formula:
  (29) $$p_{i}\propto DP\left(i\right)p\left(u_{t}^{k};l_{i}\right)p\left(z_{t}^{k};X_{t},L_{i}\right).$$

That is, the product of the DP prior and the likelihood of the observation $\left(u_{t}^{k},z_{t}^{k}\right)$. $p\left(u_{t}^{k};l_{i}\right)$ is given by the mathematical model of the semantic label. Please see the Equations (12) and (15) in [17] for the detail. $p\left(z_{t}^{k};X_{t},L_{i}\right)$ is given by the RGB-D observation model of the quadrics. According to whether it is a complete constraint model or a partial constraint model, select the corresponding formula (17) or formula (20). Then assign $y_{t}^{k}$ to objects with maximum likelihood:

(30) $$y_{t}^{k}=\arg\underset{i}{\max}p_{i}.$$

3. Update the object label: fix the data association $y_{t}^{k}$, update the posterior parameters of the Dirichlet distribution of the object label. Intuitively, the category with the most observations will be the category of the object.
4. Optimize poses and object parameters: fix data association to solve the maximum likelihood estimation of robot poses X and objects L. At this point, we have transformed it into a standard SLAM problem, so we can use formula (22) derived in the previous section to solve it.
5. Filter out false positives: treat those objects with observation number less than $n_{o}$ as false positives and filter out them. We set $n_{o}=5$ in our experiments.

## 4. Experiments

**Datasets.** To evaluate the algorithm’s performance in different scenarios, we ran experiments on the ICL-NUIM dataset [18], the TUM-RGB-D dataset [19], and an author-collected dataset recorded by a mobile robot in a home-like environment as in Table 2. The public dataset uses a handheld camera trajectory. The trajectory of ICL-NUIM covers two scenes of home and office, and the TUM-RGB-D dataset provides a total of six scenes, including three offices, one desktop and two human-made scenes. We recorded the real mobile robot dataset in a home-like environment. It contains more linear motion modes and small viewing angle observations, which can test the algorithm’s performance on the mobile robot more realistically.

Baselines. During the experiment, we chose three baselines to comprehensively evaluate the performance of our algorithm: ORB-SLAM2 [20], QuadricSLAM [15], and a point-model object SLAM [17]. ORB-SLAM2, as a state-of-the-art SLAM method based on feature points, can adequately represent the performance of conventional SLAM. We use ORB-SLAM2 with loop closures disabled as ORB-VO, and input it as odometry to three object-level SLAM algorithms to measure the improvement on trajectory accuracy after adding object landmarks. The point-model object SLAM [17] (NP for short) is a novel object-level SLAM based on a nonparametric pose graph, which uses points as an object model. This paper will compare the advantages of the quadrics model in modeling objects with it. To verify the proposed RGB-D camera-ellipsoid observation models, we also chose the state-of-the-art QuadricSLAM as a baseline. It is a monocular SLAM algorithm based on a monocular quadric observation model. Since it does not solve the data association, we will use the same association result as ours in the experiment to verify the mapping effect and localization accuracy.

The three object-level SLAM algorithms all use state-of-the-art YOLOv3 [13] to obtain object detection during the experiment. It is trained on the Coco dataset [44] and can recognize more than 80 everyday objects. We select a keyframe every ten frames in each dataset. This frequency can ensure sufficient observation constraints and avoid redundant observations.

Evaluation benchmarks. To adequately measure the SLAM algorithm’s localization accuracy, we use the absolute trajectory error to evaluate the estimated trajectory compared with the ground-truth trajectory. We calculate the root mean squared error (RMSE) as the metrics. The construction of a semantic map is the focus of this paper. The conventional SLAM algorithms generally use qualitative evaluation for mapping or reflect it in the localization accuracy. To fully evaluate the effectiveness of mapping, we propose the following metrics as benchmarks. The Trans(m) measures the center distance between the estimated object and the ground-truth object. The Shape evaluates the object’s occupied space. To remove the influence of translation and rotation, we will first translate both estimated object and ground-truth object to the origin, align their rotation axis and finally calculate the Jaccard distance (1-Intersection over Union) between their circumscribed cuboids. For the objects in Table 1 with dominant directions, Rot(deg) evaluates the minimum rotation angle required to align the estimated object’s three rotation axes with any axis of the ground-truth object to a straight line. For a trajectory, the above metrics are the average values of all correctly instantiated objects’ evaluation results. Since the baseline NP’s point model does not have the concept of rotation and occupied space, we only evaluate Trans(m) for it.

We also pay attention to the proportion of correctly instantiated objects in the map, and the instantiation success rate. We use the precision to measure the ratio of the successfully instantiated objects $n_{sins}$ and the total instantiated objects $n_{ins}$. The recall measures $n_{sins}$ and the total objects in the trajectory $n_{total}$. Since *P* and *R* trade each other, we use *F*1 as a comprehensive value that fully considers the two.

(31) $$P=\frac{n_{sins}}{n_{ins}},$$

(32) $$R=\frac{n_{sins}}{n_{total}},$$

(33) $$F1=\frac{2PR}{P+R}.$$

In actual experiments, we count objects in the trajectory with at least three observations of object detection in $n_{total}$. We define that when an estimated object’s semantic label is consistent with the ground-truth object, and the center distance is within 0.3 m, it is considered a correctly estimated object. Only correctly estimated objects will participate in the evaluation of Trans, Rot, and Shape.

The precision and recall comprehensively consider the effect of mapping and data association. When the mapping effect is poor, and the object is too far from the ground-truth, it will reduce the precision and recall. When the data association is chaotic, generating multiple instances from observations that originally belong to one object, it causes the precision to decrease. When different objects’ observations are wrongly associated to one object, recall will decrease. There is a trade-off between the precision and recall. By adjusting the false positive filtering parameter γ of the system, we can adjust the precision and recall. In actual application, identifying false positive objects may introduce wrong loops and affect the entire system. Therefore, we set up stricter γ conditions (such as more observations of objects) to get higher precision.

The above metrics can quantitatively reflect the effect of the overall algorithm in localization and mapping. We also qualitatively compare the mapping results with two object SLAM baselines to show the algorithm effects.

### 4.1. ICL-NUIM Dataset Experiment

The ICL-NUIM virtual dataset provides the rendering results of a home and an office scene. The home scene includes everyday indoor objects such as sofas, chairs, TVs, tables, vases, while the office scene includes monitors, tables, cabinets, etc. The experiment tends to verify the performance of mapping and localization in a room-scale scenario.

ICL-NUIM provides ground-truth trajectories to evaluate estimated trajectory accuracy. Although it is a virtual dataset, both the RGB and Depth images contain simulated Kinect2 camera noise to reflect the robustness of the algorithm to actual noise. Since the dataset does not give the objects’ poses and shapes in the scenes, we manually annotated the ground-truth parameters of the objects based on the ground-truth point cloud model of the dataset. The ground-truth point cloud model will also be displayed on the qualitative mapping result as a reference.

### 4.2. ICL-NUIM Dataset Experimental Results

**Figure 10:**
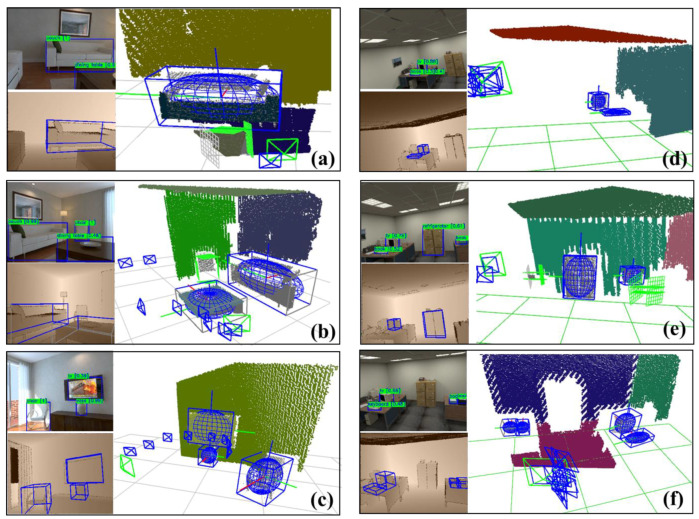
Complete and partial constraints of the ICL-NUIM dataset. (**a**–**c**) The home scene; (**d**–**f**) the office scene. In each figure, the RGB image on the upper left side indicates object detection, and the depth image indicates the projection of the estimated ellipsoid’s circumscribed rectangle into the image. The right image visualizes the complete observation model’s ellipsoids and their circumscribed rectangle, the partial observation model’s constrained planes, and the segmented Manhattan planes. The occluded constraint planes judged by the algorithm are marked in gray. Note that the camera internal parameter $f_{x}$ of the ICL dataset is inverse, so the object on the left side of the image is on the right side when visualized in 3D space. We recommend reading in color.

Figure 10 demonstrates the effects of the complete observation model and the partial observation model in the dataset and the extraction of the Manhattan planes. In the home scene, the trajectory is sometimes too close to objects, so there are many occlusions of objects. When the observation of the sofa is complete, the algorithm extracts a well-fitting ellipsoid as in Figure 10a. When the image boundary occludes part of the sofa, the algorithm marked the occluded part as invalid, as shown in gray in Figure 10b. The table in Figure 10a,b selected the complete model and the partial model in different situations, and filtered the occlusion constraint planes, which marked gray in the figure. In Figure 10b, the lower part of the chair is occluded by the table, making $P_{e}$ smaller, so the algorithm activated the partial constraint model instead. The observation model proposed in this paper can maximize observation information rather than directly eliminate it. Figure 10c shows that the algorithm extracts complete ellipsoids for TV, chairs, and vases.

Figure 10d–f demonstrates the effect in the office scene. Compared with the home scene, the trajectory is farther away from the objects, which brings difficulties to the point cloud segmentation. We can see from the figure that the algorithm has good adaptability to common office objects such as books, monitors, keyboards, as shown in Figure 10e. When the object is too small to extract the point cloud, it will switch to partial constraints.

The quantitative results of the ICL-NUIM dataset are shown in Table 3. We found that our algorithm’s trajectory accuracy in the home and office scenarios is slightly improved by 6.0% and 6.6%, respectively, compared to ORB-SLAM2. We believe that the improvement of trajectory accuracy comes from two aspects: (1) object features such as sofas, TVs, displays, have apparent dominant directions, providing reliable and precise constraints; (2) home/office scenes have walls, floors, ceilings, etc. Those low-texture fragments low down the accuracy of ORB-SLAM2, and the constraints of objects add more information to the system. In other object-level baselines, NP and QuadricSLAM also have a small improvement to ORB-SLAM2.

The evaluation of mapping is shown in Table 4. Due to the RGB-D observation models, our algorithm has achieved better translation, rotation, and shape metrics than QuadricSLAM. In terms of the object’s orientation, due to the estimation based on the support relationship and the dominant direction of the object surface, it is significantly improved compared to QuadricSLAM, which only constrains the object based on the bounding boxes. In the office dataset, we achieved a lower object translation error than QuadricSLAM. However, there is a slight increment compared with NP. We think it comes from the error of point cloud segmentation as the camera is far away from the objects in the ICL-Office dataset. We will give a more detailed discussion in Failure Cases. In the accuracy and recall rate, ours achieved advantages over baselines.

Table 5 shows the number of semantic objects in the dataset. We successfully initialized 73% and 67% of objects in the home and office dataset. Compared with the baselines shown in Table 5, ours achieved a better precision and recall of mapping. The precision and recall in the office scene are lower than the home dataset. We think the error comes from its trajectory, which is far from objects, as shown in Figure 10d–f. It brings challenges to object detection and the point cloud segmentation. Due to the small number of valid observations, some objects have not reached the initialization condition. We believe that a reasonable selection of the object-oriented observations in actual applications will bring more improvement.

**Figure 11:**
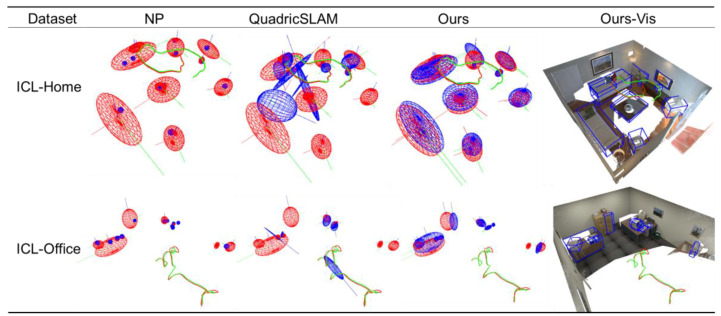
Qualitative mapping results of the ICL-NUIM dataset. It visualizes the estimated objects in blue and the ground-truth objects in red. It visualizes the estimated trajectory in green and the ground-truth trajectory in red. Ours-Vis visualizes the background as a reference and visualizes the circumscribed cuboids of the estimated ellipsoids in blue.

Figure 11 shows the qualitative mapping results. The NP algorithm restores the center point of each object based on the point model. In contrast, the algorithm based on the quadric model restores the orientation and occupied space beside the object center, providing more information for mobile robot navigation. QuadricSLAM is based on the observation model of a monocular camera. An ellipsoid needs more than three observations to converge, and the observations require significant view angle changes. In the ICL-NUIM dataset, the camera moves in a circle around the room. Some objects have too little observation, and the changing angle of view is small, resulting in weak convergence of the axis length of the object along the observation direction, and some objects fail to appear on the map due to initialization failure. Due to the RGB-D observation model, ours restores the center and shape of the object better. Ours only needs one complete observation to initialize the objects in the object map, which instantiates more objects than QuadricSLAM. Due to the statistical histogram proposed in this paper, the orientations of objects with dominant directions such as sofas, TVs, chairs, and tables are well estimated. In Ours-Vis, we visualized the point cloud map of the dataset as the background and visualized the estimated ellipsoid’s circumscribing cuboid. We can see that the object estimated by ours can fit the outer contour of the real object, which shows that although the quadric surface is a rough object model, it can meet the geometric information requirements of the robot navigation.

### 4.3. TUM-RGB-D Dataset Experiment

Compared with virtual datasets, real environments face multiple challenges such as sensor noise, illumination changes, motion blur, and occlusion. The TUM-RGB-D dataset provides the trajectory generated by a handheld Kinect recording in real scenes. We selected eight trajectories covering different desktops and low texture scenes for experimental evaluation. The dataset does not provide a ground-truth point cloud. To annotate ground-truth objects parameters to evaluate the mapping effect, we input the ground-truth trajectory and RGB-D data provided by the dataset into ElasticFusion [45]. ElasticFusion optimizes input data and obtains an accurate point cloud map. Then we annotate ground-truth objects based on the map. The point cloud mapping result will also be displayed on the qualitative mapping result as a reference.

### 4.4. TUM-RGB-D Dataset Experimental Results

**Figure 12:**
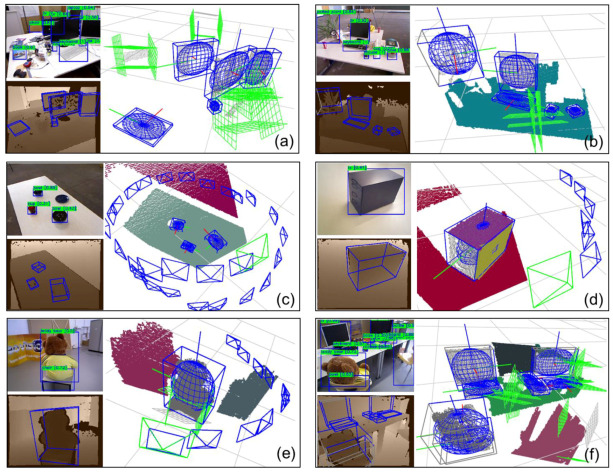
Complete and partial constraints of the TUM-RGB-D dataset. (**a**) fr1_desk; (**b**) fr2_desk; (**c**) fr2_dishes; (**d**) fr3_cabinet; (**e**) fr3_teddy; (**f**) fr3_long. Detail description can be found in Figure 10.

Real datasets bring more challenges than virtual datasets. As shown in Figure 12, the larger objects, such as monitors, teddy bears and cabinets, are estimated to be complete, while some small objects, such as cups and books, are challenging to generate complete constraints due to the small number of point clouds. Also, there are holes produced by the black display, making the point cloud of the object incomplete. In these cases, the complete constraint’s evaluation function will help the algorithm switch to partial constraints. Flexible switching of complete constraints and partial constraints enables our algorithm to maximize the use of observation information.

The experimental results of trajectory accuracy are shown in Table 6. Our algorithm can significantly improve trajectory accuracy in low-texture scenes such as fr3_dishes and fr3_cabinet, reaching an improvement of 53% and 32% compared with ORB-SLAM2, respectively. ORB-SLAM2 is difficult to obtain enough feature points to achieve robust pose estimation in low-texture scenes, where the object landmark reflects its superiority. However, some desktop scenes are cluttered and bring difficulties to data association, making the accuracy improvement small. While ORB-SLAM2 has achieved very high accuracy due to its complete loop closures, the trajectory accuracy in those desktop scenes is not significantly improved. In general, our algorithm has achieved a better accuracy improvement than NP and QuadricSLAM.

We also noticed that, in some datasets, such as fr2_desk and fr3_long, when we turned off loop closures as ORB-VO, we achieved even better accuracy than ORB-SLAM2. We think it is the error introduced by the wrong loop closures or inaccurate loop constraints.

**Figure 13:**
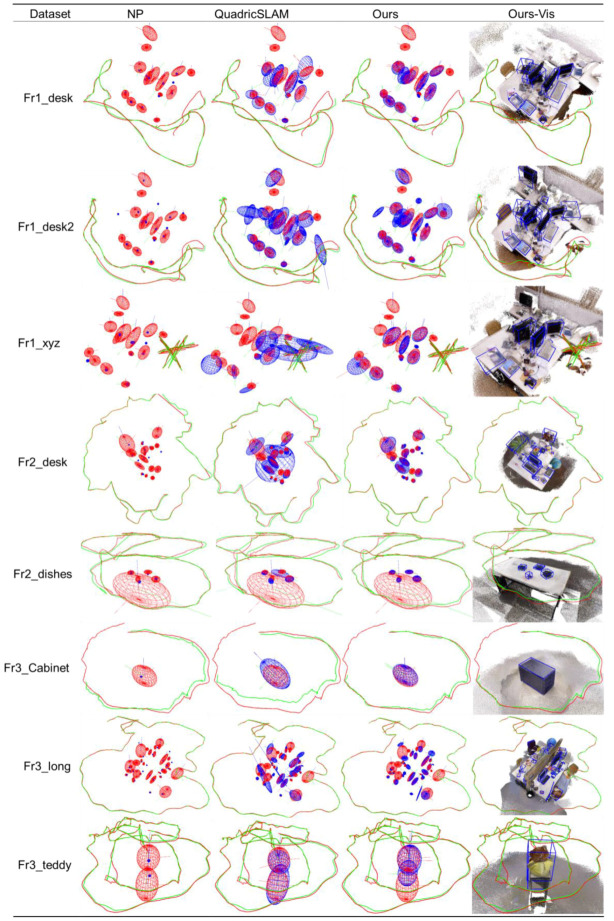
Qualitative mapping results of the TUM-RGB-D dataset. Detailed descriptions in Figure 11.

For the mapping effect shown in Table 7 and Figure 13, the proposed algorithm has distinct advantages over the baselines. We obtained the best Trans, Rot, and Shape metrics on most trajectories. Ours achieved 6.0 cm translation, 8.6 degrees rotation, and 60% IoU on average, which increased by 21%, 77%, and 40%, separately compared with QuadricSLAM. The center of the object has a 22% increase compared with the point model-based NP. Specifically, ours gets better rotation and shape compared with QuadricSLAM in the fr3_teddy dataset, while the translation is not as accurate as it in the fr3_teddy dataset. We summarize two possible reasons: First, as the camera is too close to the teddy bear, the center of the objects along the camera is not accurately estimated. Second, the trajectory is travelling around a circle, which generates large view angle changes for the monocular-based QuadricSLAM to converge.

Table 8 counts the number of all trajectory objects in the TUM-RGB-D dataset, covering a total of 100 objects. The cluttered scenes on the desktop are challenging for data association, as objects are very close to each other, and nearby objects have the same semantic label. Ours successfully instantiated 79% of them and achieved 78% precision, which is comparable with NP. Considering that the quadric observation’s data association is more complicated than the point model, experiments proved the effectiveness and the robustness of the nonparametric data association

Algorithm with the quadrics model. There is still much room for improvement. In the experiments, we found that the uncertainty of semantic labels caused by object detection caused one object to be instantiated into multiple objects. As quadrics models objects’ occupied space, we can further improve the association’s accuracy based on reasonings such as “objects cannot be overlapped” in future work.

From the qualitative experimental results of Figure 13, we list each trajectory’s experimental results in detail. It is evident from the qualitative experiment that the quadrics model contains more object information than the point model. Ours-Vis shows that the ellipsoids constructed by our algorithm fit the objects well, especially for objects with apparent dominant surface directions, such as monitors, keyboards, books, and cabinets. The object constraints serve as useful supplements to feature-based landmarks, especially under low-texture scenes.

### 4.5. Real Mobile Robot Experiment

**Figure 14:**
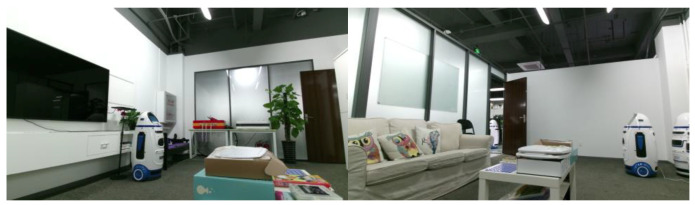
A home-like environment for a real mobile robot.

To verify the algorithm’s performance on the trajectory of a real mobile robot, we used a Turtlebot3 robot equipped with a Kinect2 to record in a real home-like environment, which includes a total of 10 common indoor objects such as televisions, sofas, beds and potted plants as in Figure 14.

Compared with ICL-NUIM and TUM-RGB-D dataset, the trajectory of a real mobile robot is more challenging. The observed object tends to maintain a small observation angle change in the vertical and pitch directions and fewer valid observations. This experiment can better verify the performance of the algorithm for mobile robot navigation than the public dataset.

Considering that the ground-truth trajectory data cannot be obtained, this experiment focuses on verifying the object-level mapping effect on the mobile robot. We build a point cloud map based on the trajectory of ORB-SLAM2 with multiple loop closures and manually annotate the poses and shapes of objects in the map as ground-truth. This experiment will focus on comparing the mapping effect with the two object-level SLAM baselines.

### 4.6. Real Mobile Robot Experimental Results

**Figure 15:**
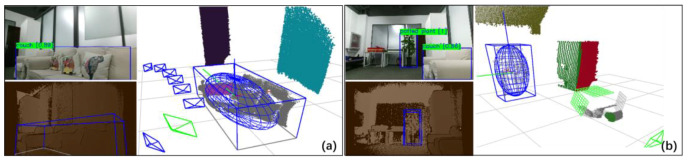
Complete and partial constraints of a mobile robot in the home-like environment. (**a**) a sofa with limited observation angle; (**b**) a sofa occluded by another one. Detailed description can be found in Figure 10.

Figure 15 demonstrates the challenge of this dataset. For example, when the mobile robot passes by the couch, limited by the observation angles, the point cloud of the couch is incomplete. There are two couch observations in the figure. The first one has enough information to estimate a complete ellipsoid and then filter the bottom occlusion constraint plane. On the right, the observation is occluded by the previous couch, which is severely incomplete, so it activated the partial constraint model to maximize information usage.

According to the experimental data in Table 9, the algorithm proposed in this paper shows distinct advantages for a mobile robot. QuadricSLAM, based on the monocular observation model, is challenging to adapt to the mobile robot’s motion, which contains small viewing angle observations. Ours, with an RGB-D observation model, maintains stable performance. Compared with QuadricSLAM, ours improves translation, rotation, and shape by 41%, 94%, and 63%. Evaluating the rotation angle, large objects such as televisions, sofas, beds, and cabinets have successfully estimated their rotation angles and reached a high angle estimation accuracy of 2.8 degrees, which can effectively help the robot to determine the semantic orientation of the object. Compared with the NP algorithm, ours improves the translation by 46% due to the better handling of the occlusion situation.

**Figure 16:**
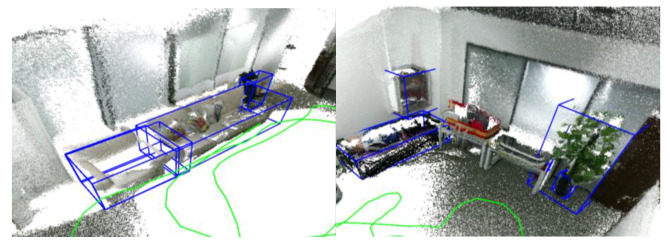
Details of the mapping results of the real-robot dataset. The algorithm proposed in this paper fits the outer contour of the actual object better. Two of the sofas successfully maintained high accuracy even under small viewing angles.

Evaluating the map’s completion, we have successfully recovered all the objects recognized by the object detection as in Table 10. Due to the efficient and robust data association, the accuracy has reached 91%, far exceeding the two object-level baselines. However, the sofa is recognized as a chair by the object detector several times under a small viewing angle as in Figure 16, making the small sofa incorrectly instantiate one more object. This problem has also appeared in previous public datasets. We will discuss it further in Failure Cases.

**Figure 17:**
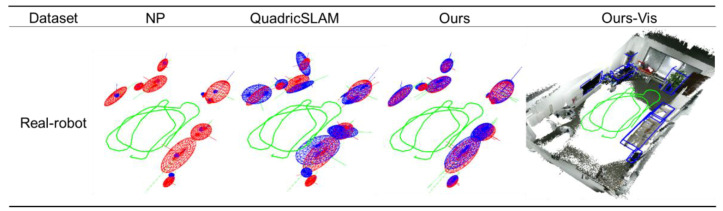
Qualitative mapping results of the real-robot dataset. Detailed descriptions in Figure 11.

Figure 17 visualizes the comparison between the result of the estimated object and the ground-truth object. Ours fits the ground-truth object better. When navigating as a semantic robot, the map can help it understand the scenes and execute semantic commands such as “Move to the TV.” The navigation based on the semantic map will be our future direction.

Table 11 shows the effect of each category of objects on the real-home dataset when we reject all the partial observations. We ignore those bounding boxes if their edges are close to the image border less than 30 pixels. When the mobile robot travels in the room, there are many partial observations because of its viewing angle. It will cause a large amount of information loss if we reject all of them directly. Especially, all the observations on the couches are partial. Overall, the introduction of partial constraints has significantly improved the object translation accuracy.

### 4.7. System Modules Analysis

We showed the orientation estimation results of three representative types of objects in three datasets in Table 12. Those objects with a dominant direction, such as the couch, TV, monitor, and cabinet, show high accuracy. While those objects are flat and have small areas along the *Z*-axis, such as book and keyboard, get lower accuracy. After optimization, the accuracy of all objects increases. As for the application for indoor mobile robot navigation, small objects such as books and keyboards are movable by human beings. We pay more attention to the objects that are large and stable to serve as reliable landmarks.

We evaluate the number of valid and invalid constraint planes for each dataset’s representative trajectory in Table 13. The occlusion of image edges causes invalid constraint planes. We use Precision and Recall to evaluate the ability to detect them. After filtering, the percent of invalid planes has reduced to around 0.3–2.5%. For the remaining outliers, we use the Huber loss in the optimization to low down their influence.

### 4.8. Failure Cases

Some situations that are difficult to deal with in the experiments have been discovered, which guides our future work. The errors mainly come from two aspects:

1. Wrong point cloud segmentation. This error mainly comes from the point cloud processing of the complete constraint model. For example, as potted plants have too thin lines, the Euclidean clustering produces only part of the plants. Due to the simple supporting planes judgment method, some objects on the ground are mistaken for being located on the desktop, so their bottom point cloud is filtered, resulting in errors. Most of the problem of point cloud segmentation can be turned into a partial constraint through the judgment of $P_{e}$, and some of the wrong cases that meet the requirements will introduce errors to the algorithm.
2. Wrong object detection. We used the YOLOv3 object detector and did not conduct targeted training for the indoor environment. Therefore, there is confusion in the detection of some objects. The object detection treats the right half part of the cabinet in the ICL-Office scene as an object most of the time. Small objects produce semantic confusion, resulting in low precision in the scene. For example, the monitors are wrongly detected as laptops several times. When there are many wrong labels, it will confuse the data associations to generate extra instances and low down the precision.

Therefore, the existence of the above problems inspires our future work. First, introduce a more robust object–support relationship judgment module, e.g., judging the relationship between the object and the supporting plane in global reasoning. Second, introduce relationships between objects, such as “objects cannot be overlapped”, to obtain a more reasonable data association. Finally, train the object detector on the indoor scene dataset to improve the detection accuracy and robustness.

### 4.9. Computation Analysis

We implement the proposed algorithm in C++, using the g2o library [46] for graph optimization, and used PCL [47] for point cloud processing. We ran the algorithm on a desktop PC with an AMD Ryzen5 3600 3.6 GHz CPU, 16 GB RedAM, and Ubuntu 16.04, as in Table 14. We present the time-consuming on the real-robot dataset as in Table 15.

It is worth mentioning that the back end runs in parallel with the front end, which can meet the mobile robots’ navigation requirements of real-time map-building and path planning. Adjusting the number of iterations can affect the convergence of object data associations, camera poses, and objects parameters. We ran iterations five times in the experiments. All the modules run on a CPU except for the object detection. The emergence of lightweight neural networks [48] recently makes object detection achieve high frame rates on a CPU, making the proposed algorithm available to run totally on a CPU in the future.

### 4.10. Memory Usage

We summarize the memory usage compared with two state-of-the-art systems in Table 16 and summarize the map storage in Table 17. Our map comprises objects with an ellipsoid consisting of nine parameters, and a semantic label comprising one parameter. We store values using the float type and store label using the unsigned char type. In a mobile robot application in a room size on the real-robot dataset, our memory usage is only 1.2 GB. And our map is only 1.63 KB. They show significant advantages compared with the dense methods.

## 5. Conclusions

This paper proposes introducing artificial objects in the indoor environment into SLAM as robust landmarks and builds an object-oriented map, which extends the traditional SLAM’s ability to understand indoor scenes. This paper proposes an object-level semantic SLAM algorithm based on RGB-D data, which uses quadrics as an object model, and compactly represents the object’s translation, orientation, and occupied space. This paper proposes two types of camera-ellipsoid observation models based on RGB-D observation data compared with the state-of-the-art monocular quadric SLAM systems. Among them, the complete observation model uses the relationship of the spatial structure plane to estimate the ellipsoid parameters from a single frame of RGB-D data. To emphasize the point cloud missing and occlusion problems, we propose a partial observation model and an evaluation function to flexibly switch between the two types of models.

The state-of-art quadric-based SLAM leaves the data association problem unsolved. This paper introduces the nonparametric pose graph and integrates it with the proposed RGB-D observation model to solve the data association in the back end robustly. Under ICL-NUIM and TUM-RGB-D datasets, and a real mobile robot dataset recorded in a home-like scene, we proved the quadrics model’s advantages. We increased the localization accuracy and mapping effects compared with two state-of-the-art object SLAM algorithms. Semantic navigation based on the object-level map, a more robust module to find the supporting planes of objects, and global scene understanding are valuable future work.

## Figures and Tables

**Table 1:** Object semantics of dominant direction’s validity. Valid objects have dominant directions on their surface. We activate the orientation constraints of the *X*-axis and *Y*-axis for them.

Objects Type	Semantic Labels
Invalid Objects	Potted Plant, Bottle, dishes, etc.
Valid Objects	TV monitor, Sofa, Chair, etc.

**Table 2:** The datasets in detail.

Datasets	ICL-NUIM	TUM-RGB-D	Real-Robot
Distance	0.1–6 m	0.1–3 m	0.2–6 m
Fps	30	30	5
Resolution	640 × 480	640 × 480	960 × 540
Sensor	Virtual	Kinect	Kinect2
Motion	Hand-held	Hand-held	Wheeled robot

**Table 3:** Trajectory RMSE(m) of the ICL-NUIM dataset.

Dataset	ORB-SLAM2	ORB-VO	NP	QuadricSLAM	Ours
Home	0.026589	0.026981	0.0257671	0.0251146	**0.0249958**
Office	0.044782	0.043508	0.0435524	0.0431719	**0.0418455**

**Table 4:** Quantitative mapping results of the ICL-NUIM dataset. *P*/*R*/*F* refers to precision, recall and *F*1.

Dataset	Items	NP	QuadricSLAM	Ours
Home	Trans/Rot/Shape	0.1162/-/-	0.1076/47.9/0.588	**0.1057/2.1/0.352**
	P/R/F	0.70/0.64/0.67	0.30/0.27/0.29	**0.80/0.73/0.76**
office	Trans/Rot/Shape	**0.0990**/-/-	0.1945/44.7/0.768	0.1230/**6.5/0.484**
	P/R/F	0.46/**0.67**/0.55	0.43/0.33/0.38	**0.55/0.67/0.60**

**Table 5:** Objects counts of the proposed algorithm in the ICL-NUIM dataset.

Dataset	#Total Objects	#Successfully Instantiated Objects	#Instantiated Objects	Precision	Recall
Home	11	8	10	0.8	0.73
Office	9	6	11	0.55	0.67

**Table 6:** Trajectory RMSE(m) of the TUM-RGB-D dataset.

Dataset	ORB-SLAM2	ORB-VO	NP	QuadricSLAM	Ours
Fr1_desk	**0.015148**	0.015768	0.0158652	0.0233906	0.0155677
Fr1_desk2	0.021463	0.022177	0.0220766	0.0219917	**0.0210426**
Fr1_xyz	0.009399	0.009878	0.00893603	0.00900908	**0.00893056**
Fr2_desk	0.01068	**0.009786**	0.010596	0.0673168	0.0100293
Fr2_dishes	0.04756	0.047644	0.0455002	0.0348168	**0.0224029**
Fr3_cabinet	0.077745	0.057812	0.0888183	0.118528	**0.0526973**
Fr3_long	0.013555	**0.009691**	0.0137451	0.0145042	0.0111203
Fr3_teddy	0.016103	0.016179	0.0160123	0.0209619	**0.0159513**

**Table 7:** Quantitative mapping results of the TUM-RGB-D dataset.

Dataset	Items	NP	QuadricSLAM	Ours
Fr1_desk	Trans/Rot/Shape	0.0637/-/-	0.0633/38.0/0.853	**0.0372/8.0/0.405**
	P/R/F	0.85/**0.79**/0.81	0.85/**0.79**/0.81	**0.92/0.79/0.85**
Fr1_desk2	Trans/Rot/Shape	0.0576/-/-	0.0778/35.0/0.689	**0.0574/8.5/0.448**
	P/R/F	**0.72/0.93/0.81**	0.67/0.86/0.75	0.68/**0.93**/0.79
Fr1_xyz	Trans/Rot/Shape	0.0690/-/-	0.1341/47.9/0.850	**0.0536/8.7/0.411**
	P/R/F	**0.80/0.57/0.67**	0.60/0.43/0.50	**0.80/0.57/0.67**
Fr2_desk	Trans/Rot/Shape	0.0492/-/-	0.0825/26.8/0.549	**0.0314/12.8/0.377**
	P/R/F	0.59/0.81/0.68	0.64/**0.88**/**0.74**	**0.65**/0.81/0.72
Fr2_dishes	Trans/Rot/Shape	0.0323/-/-	0.0415/-/0.538	**0.0201/-/0.317**
	P/R/F	0.80/0.80/0.80	0.80/0.80/0.80	0.80/0.80/0.80
Fr3_cabinet	Trans/Rot/Shape	0.0799/-/-	0.0676/40.7/0.544	**0.0421/2.0/0.150**
	P/R/F	1.00/1.00/1.00	1.00/1.00/1.00	1.00/1.00/1.00
Fr3_long	Trans/Rot/Shape	0.1033/-/-	**0.0688**/34.7/0.696	0.0793/**15.2**/**0.600**
	P/R/F	**0.93/0.84/0.89**	0.79/0.72/0.75	0.86/0.78/0.82
Fr3_teddy	Trans/Rot/Shape	0.1637/-/-	**0.0722**/40.9/0.556	0.1604/**4.8**/**0.454**
	P/R/F	1.00/1.00/1.00	1.00/1.00/1.00	1.00/1.00/1.00
Average	Trans/Rot/Shape	0.0773/-/-	0.0760/37.7/0.659	**0.0602/8.6/0.395**
	P/R/F	**0.79/0.81/0.80**	0.73/0.75/0.74	0.78/0.79/0.79

**Table 8:** Objects counts of the proposed algorithm in the TUM-RGB-D dataset.

Dataset	#Total Objects	#Successfully Instantiated Objects	#Instantiated Objects	Precision	Recall
Fr1_desk	14	11	12	0.92	0.79
Fr1_desk2	16	15	22	0.68	0.93
Fr1_xyz	14	8	10	0.8	0.57
Fr2_desk	16	13	20	0.65	0.81
fr2_dishes	5	4	5	0.8	0.8
Fr3_cabinet	1	1	1	1	1
fr3_long	32	25	29	0.86	0.78
fr3_teddy	2	2	2	1	1
Total	100	79	101	0.78	0.79

**Table 9:** Quantitative mapping results of the real-robot dataset.

Dataset	Items	NP	QuadricSLAM	Ours
Real-robot	Trans/Rot/Shape	0.1428/-/-	0.1316/43.5/0.633	**0.0771/2.8/0.237**
	P/R/F	0.82/0.9/0.86	0.73/0.80/0.76	**0.91/1/0.95**

**Table 10:** Objects counts of the proposed algorithm in the real-robot dataset.

Dataset	#Total Objects	#Successfully Instantiated Objects	#Instantiated Objects	Precision	Recall
Real-robot	10	10	11	0.91	1.0

**Table 11:** Object translation accuracy when rejecting all partial observations. “No partial” and “#ob” show the observation numbers and the translation error (m) when rejecting all partial observations. “All” and “#new ob” show the observation numbers and the translation error (m) when adding valid constraint planes of partial observations. Since the potted plant and couch have two instances, we combined their observations number and give their average translation.

	TV	Chair	Couch	Plant	Cabinet	Bed	Vase	Bottle	Average *
#ob	40	58	0	302	78	10	44	110	92
#new ob	57	107	257	409	93	40	81	129	131
no partial	0.088	0.135	-	0.141	0.032	0.172	0.057	0.166	0.113
all	0.089	0.092	0.160	0.129	0.028	0.026	0.056	0.031	0.065

* To compare the two situations fairly, we do not include Couch in the Average as “no partial” has no Couch observations.

**Table 12:** Analysis of the histogram-based orientation estimation. We show complete observations number of objects in #OB. We show the average and standard deviation of Rot(deg) among all the observations in the average and standard classes and show the optimized value in the optimized class.

	ICL-Home			Fr1_desk			Real-Robot		
Class	Couch	Chair	TV	Monitor	Book	Keyboard	TV	Couch	Cabinet
#OB	32	15	11	77	51	28	5	12	7
Average	1.72	7.39	1.63	6.21	16.24	19.57	1.62	4.52	3.06
Std	0.59	10.96	1.27	4.25	13.43	12.62	1.11	1.06	1.88
Optimized	1.40	1.34	1.21	5.95	3.26	4.32	0.24	3.69	0.30

**Table 13:** Analysis of invalid constraint planes detection. We detect and filter invalid constraint planes in both the complete observation model and the partial observation model.

	Dataset	ICL-Home	Fr1_desk	Real-Robot
Constraint Planes	Total	1620	1540	1174
	Valid	1354	1480	1056
	Invalid	266	60	118
Invalid Detection	True positive	262	44	91
	Positive	280	44	94
	False negative	4	16	27
	Precision	0.94	1.00	0.97
	Recall	0.98	0.73	0.77
Invalid Percent	Origin	16.4%	3.9%	10.1%
	After filtering	0.3%	1.1%	2.5%

**Table 14:** Algorithm running environments.

Components	Items	Components
Computation Framework	CPU	AMD Ryzen5 3600 3.6 GHz
	GPU	Nvidia 1660Super
	RAM	16 GB
Software Development Platforms	Operating System	Ubuntu 16.04
	Language	C++
Libraries	Graph Optimization	G2O
	Point Cloud Processing	PCL

**Table 15:** Algorithm time-consuming analysis.

Modules	Main Components	Runtime (ms)
Plane Module	Plane Segmentation	9.2
Object Detector	YOLOv3	33
Ellipsoid Extraction	Point Cloud Segmentation	73.5/object
	Normal Voter	8.3/object
	Total	82/object
Per Frame	Average	183.5
	Std	169.5
Back End	Data Association	26.3/iteration
	Graph Optimization	433.7/iteration

**Table 16:** Algorithm memory usage compared with ORB-SLAM2 and ElasticFusion.

	ElasticFusion	ORB-SLAM	Ours
Memory Usage	7.6 GB	2.5 GB	1.2 GB

**Table 17:** Algorithm map storage in three datasets.

Trajectory	Object Num	Map Size
ICL-Home	10	1.48 KB
Fr1_desk	12	1.78 KB
Real-Robot	11	1.63 KB
